# Predicting and Validating the Mechanism of Qingyi II Granules in the Treatment of Acute Pancreatitis by Network Pharmacology

**DOI:** 10.1155/2022/9536124

**Published:** 2022-12-07

**Authors:** Shuiping Ou, Li Ren, Xiaocui Zheng, Jianwen Yang, Yuhe Wang, Sen Wang

**Affiliations:** ^1^Department of Pharmacy, Affiliated Hospital of Zunyi Medical University, Zunyi 563000, China; ^2^Office of Drug Clinical Trials Institution, Affiliated Hospital of Zunyi Medical University, Zunyi 563000, China; ^3^Medicine & Technology College of Zunyi Medical University, Zunyi 563000, China; ^4^School of Pharmacy, Zunyi Medical University, Zunyi 563000, China; ^5^Department of Pharmacy, The First People's Hospital of Bijie, Bijie 551700, China

## Abstract

Network pharmacology, reverse molecular docking, and rat acute pancreatitis (AP) models were used to analyze the mechanism of protection by Qingyi II granules. The chemical components of 7 Chinese herbal medicines in Qingyi II granules were searched through the TCMSP (traditional Chinese medicine systems pharmacology database and analysis platform) database. The active ingredients were screened out in the OB (oral bioavailability) and DL (drug likeness) filters as a condition for inclusion. Then, the prediction analysis of potential targets was performed through databases. A GO (gene ontology) enrichment analysis of target proteins related to AP and KEGG (Kyoto Encyclopedia of Genes and Genomes) pathway annotation was performed using the DAVID (The Database for Annotation, Visualization, and Integrated Discovery) database. Finally, the “Herbal-Compound-Target” network was constructed using Cytoscape software. The active component structure and target name were uploaded to the Systems Dock database for reverse molecular docking. With octreotide as a positive control, Qingyi II decoction and Qingyi II granules were administered to AP rats at low, medium, and high doses. The pathological changes in the pancreas were observed using HE staining. The levels of Bcl-2, AMS, BAX, IL-2, and CASP3 in plasma were determined by an ELISA kit. Real-time PCR detected the expression of AKT1 and PIK3CA mRNA in the pancreas. The database predicted 94 active components of Qingyi II granules, 76 potential targets, and 64 signaling pathways. Twenty pathways were directly or indirectly associated with acute pancreatitis, including the TNF signaling pathway and the PI3K-AKT signaling pathway. In the reverse molecular docking experiment, the matching scores of the active components and the target were mainly between 6.0 and 7.0, with strong binding activity. Compared to the normal group, the plasma concentrations of BAX, IL-2, Bcl-2, AMS, and CASP3 in the model group were significantly increased (*P* < 0.05). Compared with the model group, the low-dose group of Qingyi II granules only significantly reduced IL-2 levels and had no effect on other indicators. The other groups could significantly reduce the levels of AMS, BAX, and CASP3 (*P* < 0.05). Compared with the model group, the octreotide group and Qingyi II granules high-dose group significantly increased the Bcl-2 level (*P* < 0.05), and there was no statistical difference in other drug-administered groups. Compared with the normal group, the expression of AKT1 and PIK3CA in the pancreas of the model group was significantly higher. Compared to the model group, the expression of PIK3CA was low in all drug-administered groups. In addition to the low-dose group, the other drug-administered groups significantly reduced the expression of AKT1. Qingyi can reduce the levels of AMS, BAX, IL-2, and CASP3 and increase the levels of Bcl-2. This mechanism may be related to the PI3K- AKT signaling pathway.

## 1. Introduction

Acute pancreatitis (AP) is a common clinical condition with rapid onset, rapid progression, and a high mortality rate that can quickly progress to severe acute pancreatitis (SAP). SAP is often accompanied by local or systemic inflammatory response syndrome and systemic multiple organ dysfunction [1, 2]. The etiology and pathogenesis of AP are complex and multifactorial pathological processes. The activation of trypsin and the self-digestion of the pancreas, pancreatic microcirculatory disturbance, inflammatory reaction, and intestinal bacterial translocation can cause AP [3, 4].

Qingyi II granules are derived from the clinical experience of the Affiliated Hospital of Zunyi Medical University. It is composed of *Rhei Radix Et Rhizoma* (Da Huang), *Paeoniae Radix Rubra* (Chi Shao), *Gardeniae Fructus* (Zhi Zi), *Aucklandiae Radix* (Mu Xiang), *Magnoliae Officinalis Cortex* (Hou Po), *Coptidis Rhizoma* (Huang Lian), *Corydalis Rhizoma* (yanhusuo), *Moutan Cortex* (Mu Dan Pi), and *Mirabilite* (Mang xiao) [5–7]. Da Huang is used as monarch medicine. It is supplemented by Chi Shao, Zhi Zi, Mu Xiang, Hou Po, Huang Lian, yanhusuo, and Mang Xiao. It is combined with various drugs to relieve the depressed liver, clearing away heat, toxic materials, analgesia, and the Tongli attack effect. It is mainly used in spleen and stomach dampness-heat type acute pancreatitis [8]. Many experimental and clinical studies show that Qingyi II granules have a good effect on AP and its complications [9–13]. Network pharmacology is a new discipline based on the theory of systems biology, which analyzes the network of biological systems and selects specific signal nodes for the design of multitarget drug molecules. Network pharmacology emphasizes multipathway regulation of the signaling pathway to improve the therapeutic effect of drugs and reduce toxicities and side effects, so as to improve the success rate of clinical trials of new drugs and save drug research and development costs [14, 15]. The network pharmacology is a kind of network based on the “drug-target-disease” interaction, using the chemical constituents known in traditional Chinese medicine, combining the existing research results, exploring the multitarget and multiway synergistic effect of the components, and visually displaying the complex action by the network [16]. Reverse molecular docking is a process that uses small molecular compounds (natural products, lead compounds, and chemical compounds) as probes to search the target database of known structures for biological macromolecules that may bind to them. Molecular complexes are formed through spatial and energy matching to predict the potential targets of drugs [17–19]. The holistic and comprehensive characteristics of this research are consistent with the principle of synergy between Chinese medicine and its compound multicomponent, multichannel, and multitarget [20, 21]. Therefore, this subject combined with the network pharmacology technology can predict the potential of active components, active targets, and the pathways of Qingyi II granules and further verify the active components and targets by using the reverse molecular docking technique. The ELISA method and the PCR method are used for the experimental verification of the predicted results. They provide a reference for the development and clinical application of Qingyi II granules.

## 2. Materials and Methods

### 2.1. Experimental Animals

Male Sprague–Dawley rats, weighing 210–250 g, were purchased from Changsha TianQin Biotechnology Co., Ltd., license number: SCXK (Xiang) 2014–0011. This protocol was approved by the Animal Care and Use Committee of the Zunyi Medical University (2019-2-252), and all experimental procedures were in compliance with the National Institutes of Health Guide for Care and Use of Laboratory Animals.

### 2.2. Drugs and Reagents

Qingyi II granules and Qingyi II broth are homemade buds (lot number: 180228-C-0301). Hefei Semenno Biotechnology Co., Ltd, lipopolysaccharide (L2880, batch number: 028M4094V) Sigma, USA, octreotide acetate injection liquid (batch number: 180608) Taishan Pharmaceutical Co., Ltd, Animal Total RNA isolation Kit, RT EasyTM II, and Real-Time PCR EasyTM were purchased from FOREGENE. ELISA kits for Bcl-2, AMS, BAX, IL-2, and CASP3 were purchased from Shanghai Shenggong Bioengineering Co., Ltd., and anhydrous ethanol, sodium chloride, potassium dichromate, and concentrated concentration sulfuric acid were purchased from the Chengdu Branch. Long Chemical Reagent Factory, formaldehyde was purchased from Shanghai Titan Technology Co., Ltd, and xylene was purchased from Tianjin Zhiyuan Chemical Reagent Co., Ltd. Hematoxylin dyeing solution (Cat. No. BA-4097) and Yihong dyeing liquid (Cat. No. BA-4099) were purchased from Zhuhai Besso Biotechnology Co., Ltd.

### 2.3. Main Instruments

Multiskan spectrum full-wavelength microplate reader (Thermo Company, USA), tissue microtome (Leica-2016, Germany), BMJ-III type embedding machine, TSJ-II type fully automatic closed tissue dehydrator (Changzhou Zhongwei electronic instrument) Co., Ltd), BA400Digital microscope (McAudi Industrial Group Co., Ltd.), PIKORed 96 real-time fluorescence meter (American ThermoFisher Instrument Co., Ltd.), and TCA0096 thermal cycler (American ThermoFisher Instrument Co., Ltd) were used as primary instruments.

### 2.4. Acquisition of Chemical Constituents and Screening of Active

Through the databases TCMSP (https://sm.nwsuaf.edu.cn/lsp/tcmsp.php/), China Traditional Chinese Medicine Integration Database TCMID (https://www.megabionet.org/tcmid/), Taiwan Traditional Chinese Medicine Database TCM Database@Taiwan (https://tcm.cmu.edu.tw/), and other Chinese herbal medicines in the chemical composition database, we are collecting Qingyi II granules in the Rhubarb, Radix Paeoniae Rubra, Gardenia, Common Vladimiria Root, Magnolia Officinalis Rehd. Et Wils, Rhizoma Corydalis, and Cortex Moutan. All the chemical components were combined with the existing literature data for data mining and finishing, based on the chemical components contained in the TCMSP database. The chemical name and number shall be unified, and the duplicate numerator shall be deleted. By limiting the bioavailability of chemical components of traditional Chinese medicine (OB ≥ 30%) and the drug similarity (DL ≥ 0.18), [22, 23] combined with the literature review, the active components in the Qingyi II granule compound were screened.

### 2.5. The Prediction of Potential Targets of Active

The active components were imported into the TCMSP, STITCH, Target-Prediction, and Swiss Target-Prediction databases to obtain their effect targets. After the deduplication was summarized, the target name was input into Uniprot (the limited species was human and existed in rats simultaneously) and converted to a gene name. In the OMIM database, GAD database, TTD database, and PharmGKB database, input keywords “acute pancreatitis” and “Severe acute pancreatitis” to retrieve genes related to acute pancreatitis, remove duplicate and false-positive genes, and match the targets of active components. The potential target of Qingyi II granule active ingredient in the treatment of AP was obtained.

### 2.6. Construction of the Medicinal Materials-ActiveComponents-Target Network

Qingyi II granules compound medicinal materials, active components, and target data sets were constructed and introduced into the Cytoscape software. The medicinal materials, components, and targets were constructed into a ternary comprehensive network model that can reflect the medicinal materials, components, and targets using the combined function (Merge) in the software. The potential targets related to AP were introduced into Cytoscape, a construction component-potential target network.

### 2.7. Target Pathway Analysis

The potential target was imported into the biomolecular function annotation system (DAVID), the “official gene symbol” was selected, the research object was defined as human, and the KEGG pathway annotation and GO enrichment annotation analysis were performed on the AP target. According to the principle of AP correlation reported in the literature, a significant enrichment pathway (*P* < 0.05) related to AP was screened and plotted as a bar graph, and OMIC shares drew a high-level bubble map to screen the key pathways to construct a target-pathway network.

### 2.8. Molecular Docking Verification

The System Dock (https://systemsdock.unit.oist.jp/) database was used to perform molecular docking of key active components and key targets for the treatment of AP to further validate the target's reliability. The dock score of the active ingredient and the target protein was obtained by inputting the PDB ID of the target protein and introducing the structure of the active ingredient. The degree of match between the active ingredient and the target protein was judged based on the dock Score.

### 2.9. Preparation of the Rat AP Model and Grouping Administration

A total of 56 male Sprague–Dawley rats, weighing 200–250 g, were fed for one week. They were randomly divided into seven groups, eight in each group, including the control group, model group, octreotide group, Qingyi II decoction group, and Qingyi II granules in the low, medium, and high-dose groups. They were fasting for 12 h, drinking only water. AP rats were induced with intraperitoneal injections of caerulein (CAE) 60 *μ*g/kg every 1 h for 6 h, the final intraperitoneal injection of CAE, and intraperitoneal injections of lipopolysaccharide (LPS) 15 mg/kg. After successful modeling, octreotide group (100 *μ*g/kg), Qingyi II decoction group (13 g/kg), and Qingyi II granules in low-dose groups (6.5 g/kg), medium-dose groups (13 g/kg), and high (26 g/kg) dose groups, the three groups were dosed once a day for five consecutive days. Octreotide was injected subcutaneously, and the other groups were intragastrically administered. The control and model groups were given the same amount of normal saline.

Blood was collected from the abdominal aorta after the last administration, and the plasma was taken by centrifugation (5000 rpm, 10 min) and stored in a refrigerator at −20°C. After the rats died, the pancreas was quickly removed, placed in a Petri dish containing physiological saline, rinsed, and blotted dry using filter paper. The same portion of the pancreas was cut into a 10% formaldehyde solution for fixation. The gradient alcohol was dehydrated, made transparent with xylene, serially sliced with paraffin, and subjected to HE staining to observe pancreatic tissue lesions. The ELISA kit detected the levels of Bcl-2, AMS, BAX, IL-2, and CASP3 in plasma. Real-time PCR detected the expression of AKT1 and PIK3CA mRNA in the pancreas.

Statistical analysis was performed using SPSS 17.0 software. The differences were expressed as the mean ± standard deviation. A one-way analysis of variance was used between groups. The LSD test was used to compare groups with variance, and Dunnett's T3 test was used for groups with irregular variance. Dunnett's T3 test was performed for those with irregular variance. *P* < 0.05 indicated that the difference was statistically significant.

## 3. Results

### 3.1. Screening of Active Components and Targets of Qingyi II Granule in the Treatment of AP

#### 3.1.1. Active Ingredient and Target Screening

**Figure 1:**
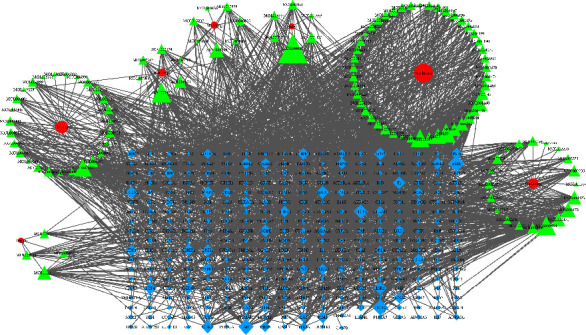
Components-target network of Qingyi II granules (red represents medicinal materials, yellow represents components, and blue represents targets).

Through the TCMSP database, 564 kinds of chemical components in Qingyi II granules were collected, including 92 kinds of *Rhubarb*, 75 kinds of *Radix Paeoniae Rubra*, 77 kinds of *Rhizoma Corydalis*, 55 kinds of *Cortex Moutan*, 107 kinds of *Common Vladimiria Root*, 139 kinds of *Magnolia Officinalis Rehd. Et Wils*, and *Gardenia* 19 kind. After collecting and removing repetition and screening by OB ≥ 30% and DL ≥ 0.18, 104 active components were obtained. According to screening rules, 12 components such as Emodin, Physcion, Geniposide, Paeonol, Magnolol, and Honokiol were initially removed, and the Chinese pharmacopeia quantitatively identified these components. The literature has reported that these ingredients have a good effect on acute pancreatitis. Therefore, these components were manually searched for further analysis, and 116 chemical components were finally obtained. A total of 116 components were entered into databases such as TCMSP and STITCH, and the target points of *Rhubarb*, *Radix Paeoniae Rubra*, *Gardenia*, *Common Vladimiria Root*, *Magnolia Officinalis Rehd*. *Et Wils, Rhizoma Corydalis,* and *Cortex Moutan* were given 107, 80, 148, 82, 136, 118, and 223, respectively. A total of 336 targets were removed from the components, including repetitive targets that did not have corresponding targets. The selected Qingyi II granules, active ingredients, and target were sequentially introduced into Cytoscape software to construct a Qingyi II granules-activeingredient-target network (Figure 1). Each edge in the figure represents the interaction between the drug and the compound or the compound and the target. The greater the effect in a network, the more edges connected to it, and the higher the degree of the node.

#### 3.1.2. Qingyi II Granules for the Treatment of AP Component-Target Network

**Figure 2:**
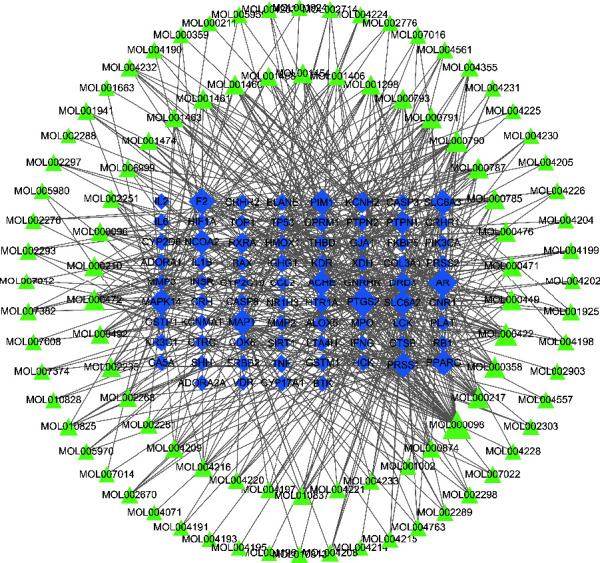
Qingyi II therapy AP component-target network (yellow represents component and green represents target).

A total of 1023 known AP-related genes were found by the OMIM, GAD, TTD, and PharmGKB databases, and a total of 1012 were obtained after deleting the duplicate genes. When 76 targets from Figure 1 were compared, 94 active components in Qingyi II granules were applied to these targets. Possible therapeutic targets in this formulation are shown. The active compounds and potential targets were constructed to construct an anti-APcompound-target interaction network (Figure 2). It contains 170 nodes (94 compounds and 76 target sites) and 561 edges.

In Figure 2, MOL000098 (Quercetin) has the highest degree (34 targets), which makes it the central node of the network, derived from Cortex Moutan, Gardenia, and Rhizoma Corydalis. This suggests that quercetin may be a key active ingredient in the efficacy of Qingyi II granules, followed by MOL000422 (kaempferol), MOL000790 (isocorypalmine), MOL000217 ((S)-Scoulerine), and MOL000472 (emodin), which had 18, 16, 16, and 15 targets, respectively. Quercetin and Kaempferol belong to flavonoids, isocordierquat and (S)-scoulerine belong to alkaloids, and emodin belongs to anthraquinone, which can inhibit the release of inflammatory factors, sterilization, and antivirus, etc [24–26]. It is indicated that flavonoids, alkaloids, and anthraquinones may be essential components of the clear Qingyi II granules. In the network, prostaglandin G/H synthase 2 (PTGS2) and androgen receptor (AR) have 39 and 38 compounds, respectively. There were 29 thrombin and acetylcholinesterase (ACHE), 27 mitogen-activated protein kinase 14 (MAPK14), peroxisome proliferator-activated receptors (PPARs), and trypsin 1 (PRSS1). Twenty-six compounds work together, and it is speculated that it may be a key target for AP treatment with Qingyi II granules, reflecting the characteristics of multiple components and multi-target interactions of Qingyi II granules.

### 3.2. Target Biological Process Analysis

**Figure 3:**
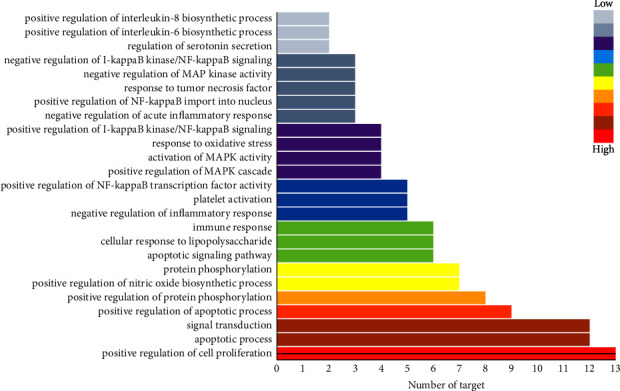
Enrichment analysis of the GO biological process of potential active ingredients in the treatment of AP.

Using the DAVID database for GO enrichment analysis (Figure 3), the results are shown in Figure 3. The results show that biological processes are closely related to the positive regulation of cell proliferation, the apoptosis process, signal transduction, and the inflammatory response, reflecting that AP pathogenesis is related to multiple biological processes in vivo. It also shows that the Qingyi compound may play a role in treating AP by improving these biological processes.

### 3.3. Target Signal Path Analysis

**Figure 4:**
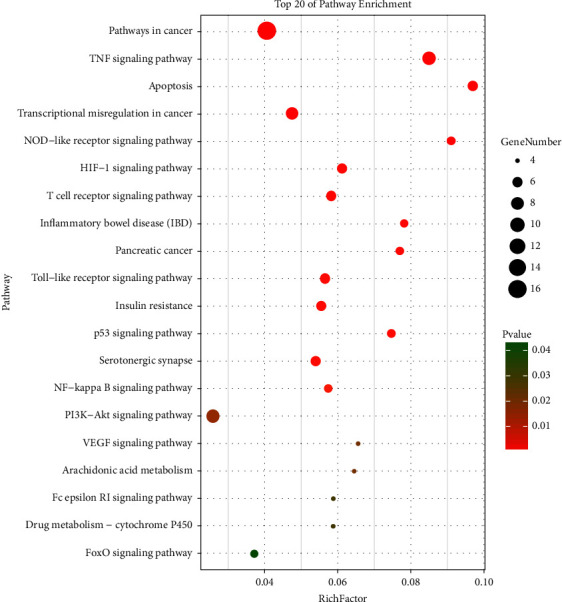
Rich distribution of differentially expressed genes pathway.

The potential targets were imported into the DAVID database for KEGG analysis, and 20 signaling pathways with a high correlation with AP were selected (*P* < 0.05), as shown in Table 1. The high-level bubble map is drawn by OMIC share, as shown in Figure 4. The enrichment degree of the differentially expressed genes in the pathway is reflected. The enrichment degree of KEGG is compared by the number of genes on the pathway, the enrichment factor (Rich factor represents the ratio of the number of genes located in this pathway entry to the total number of all genes located in this pathway entry), and *P* value. The greater the rich factor, the greater the degree of enrichment.

**Figure 5:**
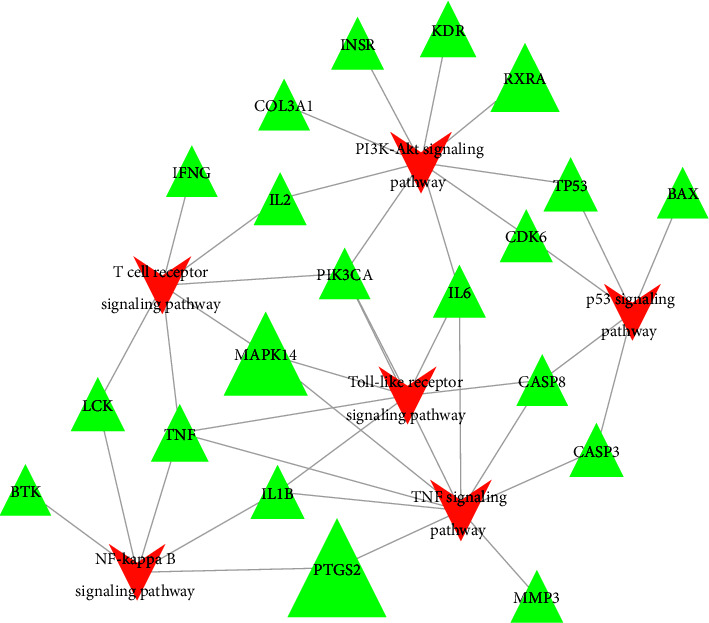
Target-pathway network (*P* < 0.05).

Six key pathways for the treatment of AP-potential targets were screened out with AP-related pathways (*P* < 0.05). The Cytoscape software was used to construct a target-path network, as shown in Figure 5. Nine potential targets are acting on the TNF and PI3K-AKT signaling pathways; six targets synergistically act on the T-cell receptor signaling pathway and the toll-like receptor signaling pathway; participating in the p53 signaling pathway; and five targets of the NF-*κ*B signaling pathway. This indicates that the Qingyi II granules may achieve their purpose of treating AP by regulating these signaling path-related genes.

### 3.4. Analysis of Molecular Docking Verification Results

The target of the previously predicted pathway is used for molecular docking with the primary active ingredient in the formulation. Aloe Aloe-Emodin, Rhein, Emodin, Physcion, Paeonol, Geniposide, and Costunolide were ligated with IL-2, Bcl-2, BAX, CASP3, AKT1, and PIK3CA, respectively. The degree of matching between the component and the target protein is determined by the docking score value. [27] The results are shown in Table 2. The score value above 4.25 indicates that there is a certain amount of binding between the active ingredient and the key target. If the value is greater than 5.0, it indicates better activity, and greater than 7.0 indicates strong binding activity. [28] From the table below, the docking score between the active ingredient and the target is higher than 4.25, mainly concentrated between 6.0 and 7.0. The results indicate that the active ingredient has a good binding activity to the target protein. This result verifies the reliability of the predicted target. These targets can be used to detect pharmacodynamic indicators and for further experimental verification.

### 3.5. Antirhinitis Active Ingredient—Target Network

**Figure 6:**
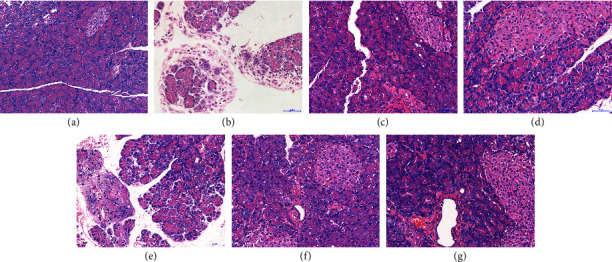
Pathological examination of rat pancreas (HE × 400).

As shown in Figure 6, edema, slight degeneration and necrosis of acinar epithelial cells, and infiltration of interstitial cells occurred in the model group when compared to the blank group, indicating that the disease in the model group changed significantly. Compared to the model group, the pathological changes in the low-dose group were not significantly improved. The positive control group, the decoction group, the middle-dose group, and the high-dose group were improved to varying degrees, suggesting the poor therapeutic effect of the low-dose group and the decoction group. The middle-dose group and the high-dose group have a certain therapeutic effect.

### 3.6. Effect of AMS, IL-2, Bcl-2, BAX, and CASP3 on Plasma in Rats

**Figure 7:**
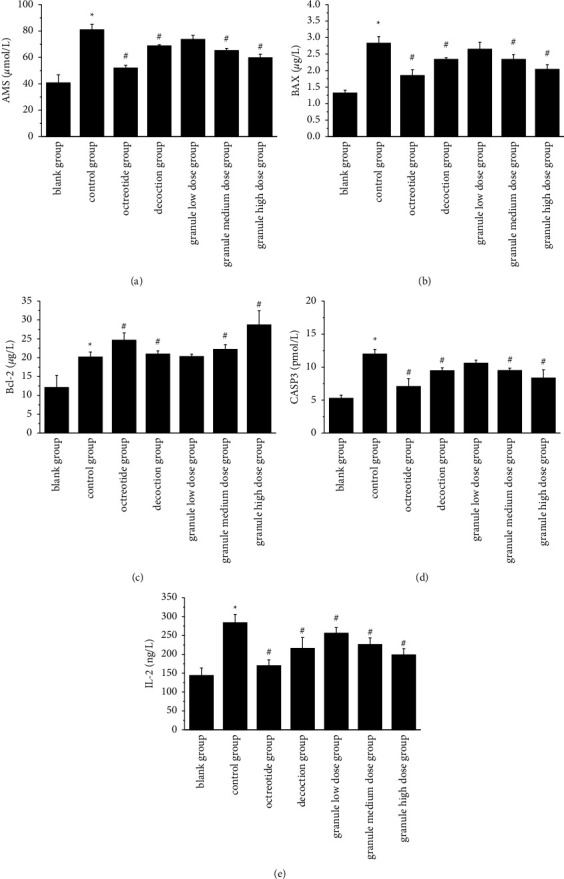
Levels of IL-2 (a), AMS (b), CASP3 (c), BAX (d), and Bcl-2 (e) in each group. Note. comparison between the model group and blank group: *∗* *P*  <  0.05; comparison between the drug administration group and model group: # *P*  <  0.05.

As shown in Figure 7, the plasma BAX, IL-2, Bcl-2, AMS, and CASP3 concentrations in the model group, were significantly higher than those in the blank control group (*P*  <  0.05). Compared to the model group, the low-dose group of Qingyi II granules only significantly reduced IL-2 levels and did not affect other indicators. Compared with the model group, except for the low-dose group of Qingyi II granules, all the other administration groups could significantly decrease the levels of AMS, CASP3, and BAX (*P*  <  0.05). Compared with the model group, the octreotide group and the high-dose group of Qingyi II granules significantly increased the level of Bcl-2 (*P*  <  0.05), but there was no significant difference in other administration groups.

### 3.7. Effects on AKT1 and PIK3CA mRNA Expression

**Figure 8:**
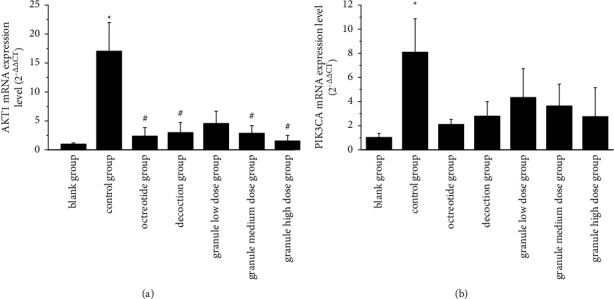
mRNA expression of AKT1 (a) and PIK3CA (b) in each group. Note. Comparison between model group and blank group: *∗* *P*  <  0.05; comparison between drug administration group and model group: # *P*  <  0.05.

Compared with the normal group (Figure 8(a)), the expression of AKT1 mRNA in the model group was significantly increased (*P*  <  0.05). There was no statistically significant difference between the low-dose group and the model group, but the expression of AKT1 mRNA in the other administration groups was significantly lower than that in the model group (*P*  <  0.05). Compared to the normal group (Figure 8(b)), the expression of PIK3CA mRNA in the model group was significantly increased (*P*  <  0.05). Compared to the model group, PIK3CA mRNA was not statistically significant in all the administration groups.

## 4. Conclusion and Discussion

Network pharmacology utilizes the analysis of pathway targets related to drugs and diseases to predict the therapeutic effect of drugs, which has the potential to improve clinical trials of new drugs. The success rate and the advantage of saving drug research and development costs [29]. By referring to the network pharmacology evaluation method guide, the data information of different platforms was collected and compared. Analyze and use this method to predict the mechanism of Qingyi II granules in the treatment of acute pancreatitis and guide subsequent experimental verification [30, 31].

In clinical practice, Qingyi II decoction is used as medicine. Although decoction is suitable for the principle and flexibility of TCM syndrome differentiation and treatment, plus or minus symptoms, it is bulky, easy to deteriorate, and inconvenient to take, carry, and store. The previous research of our research group shows that after Qingyi II decoction is changed into Qingyi II granules, it can not only ensure the curative effect but also be convenient for patients to take, carry, and store and is superior to the decoction in terms of quality control and preparation stability. The etiology and pathogenesis of acute pancreatitis are complex multifactor pathological processes. Qingyi II granules are effective in the treatment of spleen-stomachdampness-heat acute pancreatitis. However, its mechanism is not clear. The integrity and comprehensiveness of the network pharmacology research strategy are consistent with the principle of multicomponent, multichannel, and multitarget synergy of traditional Chinese medicine and its compounds [32]. It is beneficial to explain the mechanism of AP treatment systematically and comprehensively.

This study discovered that Aloe-Emodin, Rhein, Emodin, and Physcion in Qingyi II granule compounds could directly act on PIK3CA, MAPK14, or IL-2, IL-1B, IL-6, and TNF via component and target prediction. The targets of PAEONOL are BCL-2, IL-2, and TNF. Geniposide can act on BCL-2, Costunolide, BAX, and CASP3. These targets are important for predicting six signaling pathways. These six signaling pathways have been reported in the literature. A PI3K-AKT signaling pathway is downstream of the toll-like receptor pathway and the TNF signaling pathway, but upstream of the NF-*κ*B and the p53 signaling pathways in the pathogenesis of AP.

The PI3K/AKT pathway is an important information pathway in the pathogenesis of AP. Current studies have confirmed that PI3K/AKT can treat acute pancreatitis through multitarget, multipathway, and multifunction. There are three types of PI3K: I, II, and III. Among them, the most widely studied class I PI3Ks are composed of the regulatory subunit P85 and the catalytic subunit P110. PI3K is the intermediate bridge of a variety of cellular responses and plays a key role in cell apoptosis, pyroptosis, autophagy, oxidative stress, and other pathways under the influence of a variety of upstream or downstream factors. AKT, also known as protein kinase B (PKB), is a serine/threonine protein kinase composed of AKT1, AKT2, and AKT3 subtypes. Activated PI3K phosphorylates AKT at phosphorylation sites Ser473 and Thr308, and AKT activates or inhibits its downstream targets such as NF-*κ*B and mTOR to participate in the growth and survival of the body [33]. The PI3K/Akt signaling pathway is involved in the physiology and pathology of various tumors and can significantly inhibit the proliferation, migration and metastasis of cancer cells [34, 35]. It has been reported that the PI3K-AKT signaling pathway can effectively regulate the release of inflammatory factors to achieve AP prevention and treatment [36]. Therefore, the PI3K-AKT signaling pathway plays an important role in the treatment of AP by Qingyi II granules. The results of the molecular docking experiments show that there is good binding activity between the main active ingredients and the key target [37]. The reliability of the predicted target was further verified. So, we selected this pathway and the downstream targets AMS, IL-2, Bcl-2, BAX, and CASP3 for pharmacodynamic validation.

The modeling method used in this experiment is simple, and its success rate is high. There are many methods to establish the acute pancreatitis model. CAE can only induce mild acute pancreatitis. In this experiment, CAE combined with LPS was selected to establish the SAP model. After modeling, it was observed that the surface of the pancreas was dark red, with hyperemia and edema. Under the microscope, the pancreatic tissue had cell degeneration and necrosis, edema, and interstitial inflammatory cell infiltration. The serum amylase was significantly increased by abdominal aortic blood collection. The model was successfully established.

The results of the pharmacokinetics test showed that Qingyi II granules could up-regulate the expression of AKT1 mRNA, decrease the concentration of AMS, IL-2, BAX, and CASP3, and increase the concentration of Bcl-2. PI3K-AKT is an important mediator for regulating the expression of NF-*κ*B and downstream genes. Several studies have confirmed its vital role in the occurrence and development of acute pancreatitis. It is speculated that the mechanism of Qingyi II granules in the treatment of acute pancreatitis may be through inhibiting AKT1 phosphorylation, decreasing Bax, increasing Bcl-2 expression inhibiting the activation, and release of CASP3, and inhibiting apoptosis.

In conclusion, it is correct to study the mechanism of the Qingyi II granule in the prevention and treatment of acute pancreatitis using the combination of network pharmacology and molecular docking technology. This reveals the characteristics of multicomponent, multitarget, and multichannel treatment of Qingyi II granules. It also embodies the principles of integrity and systematicity in the treatment of diseases with traditional Chinese medicine. It is of great significance to explore the targeted regulation of the PI3K/AKT signaling pathway in the prevention and treatment of AP from the perspective of related targets and cell functions of the PI3K/AKT signaling pathway and traditional Chinese medicine treatment of AP, which is expected to provide new ideas for basic research and clinical treatment of AP. The synergistic effects of the multiple compounds in Qingyi II granules and the relationship between the effects of these compounds and their mechanisms in AP will be investigated in the future.

## Figures and Tables

**Table 1:** Correlated pathways of Qingyi II granules (*P* < 0.05).

Pathways	Number of genes	Pop hits	*P*
Pathways in cancer	16	393	4.45*E* − 06
TNF signaling pathway	9	106	8.11*E* − 06
PI3K-Akt signaling pathway	9	345	0.0192384
Transcriptional misregulation in cancer	8	168	0.0012338
Apoptosis	6	62	3.33*E* − 04
HIF-1 signaling pathway	6	98	0.002665
T-cell receptor signaling pathway	6	103	0.0033081
Toll-like receptor signaling pathway	6	106	0.0037439
Insulin resistance	6	108	0.0040564
Serotonergic synapse	6	111	0.0045597
NOD-like receptor signaling pathway	5	55	0.0020061
Inflammatory bowel disease (IBD)	5	64	0.0034978
Pancreatic cancer	5	65	0.0036999
p53 signaling pathway	5	67	0.0041276
NF-kappa B signaling pathway	5	87	0.0103534
FoxO signaling pathway	5	134	0.0424579
VEGF signaling pathway	4	61	0.021856
Arachidonic acid metabolism	4	62	0.0228089
Drug metabolism - cytochrome P450	4	68	0.0289965
Fc epsilon RI signaling pathway	4	68	0.0289965

**Table 2:** Molecular docking score between active components and key targets.

Components	Targets					
	IL-2	BAX	PIK3CA	Bcl-2	AKT1	CASP3
Aloe-emodin	6.700	6.884	6.680	6.082	6.766	4.588
Costunolide	5.808	6.330	6.662	5.937	6.438	4.727
Rhein	6.813	6.805	6.493	6.016	6.761	4.524
Physcion	6.864	6.943	6.348	6.086	6.426	4.692
Emodin	6.847	6.801	6.649	6.097	6.719	4.565
Geniposide	5.401	5.816	6.379	5.820	6.330	5.877
Paeonol	3.298	3.582	4.068	3.380	4.029	3.885

## Data Availability

The data included in this study is available upon reasonable request to the corresponding author.
